# The Fate of the Aorta after Coarctation Repair: Open Surgical Replacement of Descending Aorta in a High-Volume Unit

**DOI:** 10.3390/jcm13185345

**Published:** 2024-09-10

**Authors:** Ezin Deniz, Dmitry Bobylev, Heike Krüger, Jawad Salman, Alina Zubarevich, Andreas Martens, Tim Kaufeld, Bastian Schmack, Alexander Weymann, Arjang Ruhparwar, Aron-Frederik Popov, Florian Helms

**Affiliations:** 1 Division for Cardiothoracic-, Transplantation- and Vascular Surgery, Hannover Medical School, 30625 Hannover, Germany; 2 Clinic for Cardiac Surgery, University Clinic Oldenburg, 26133 Oldenburg, Germany

**Keywords:** descending aorta replacement, coarctation of the aorta, adult congenital heart disease

## Abstract

**Objectives:** Complications after aortic coarctation repair are associated with high mortality and require surgical or endovascular reintervention. For patients unsuitable for endovascular therapies, reoperation remains the only therapeutic option. However, surgical experience and up-to-date follow-up data concerning this overall rare entity in the spectrum of aortic reoperations are still highly limited. Thus, the aim of this study was to analyze the short-term outcomes and long-term survival of patients undergoing surgical descending aorta repair after previous coarctation repair in a high-volume unit. **Methods:** We present a retrospective single-center analysis of 25 patients who underwent open descending aorta replacement after initial coarctation repair. The surgical history, concomitant cardiovascular malformations, and preoperative characteristics as well as postoperative complications and long-term survival were analyzed. **Results:** The mean age at operation was 45.4 ± 12.8 years. A proportion of 68% (n = 17) of the patients were male. The most common complication necessitating reoperation after coarctation repair was aneurysm formation (68%) and re-stenosis (16%). The average time between initial repair and reoperation was 26.3 ± 9.9 years. Technical success was achieved in all the operations, while recurrent nerve damage (24%) and bleeding requiring rethoracotomy (20%) were identified as the most common perioperative complications. The one-year mortality was 0% and the overall long-term survival was 88% at 15 years. **Conclusions:** Open surgical descending aorta replacement can be performed safely and with excellent survival outcomes even in the challenging subgroup of patients after previous coarctation repair. Thus, reoperation should be considered a feasible approach for patients who are unsuitable for endovascular therapies. Nonetheless, concomitant cardiovascular anomalies and frequent preoperations may complicate the redo operation in this patient population.

## 1. Introduction

Aortic coarctation is among the most common congenital cardiovascular abnormalities accounting for 5–8% of all congenital heart diseases and making surgical coarctation repair one of the most frequently performed procedures in congenital cardiovascular surgery [1,2]. Over the last few decades, the surgical technique for primary coarctation repair has drastically changed from patch aortoplasty [3,4] and graft interposition [5] to the extended end-to-end anastomosis technique used predominantly today [5,6]. Nonetheless, with the improved overall survival of patients with congenital cardiovascular diseases, the management of long-term complications in adults after coarctation repair with one of the previously established techniques is now moving more and more into the center of attention. Here, re-coarctation as well as aneurysmatic complications and graft infections may occur as challenging late complications. The conservative management of these complications resulted in a mortality of up to 100% in earlier reports [7]. Thus, reoperation or -intervention is inevitable for complicated coarctation repairs. Concomitant congenital cardiovascular abnormalities such as bicuspid aortic valve or aortic arch anomalies, which are frequently associated with congenital aortic coarctation [8], may furthermore increase the operative and perioperative risk of redo descending aorta replacement. While endovascular therapies such as thoracic endovascular aortic repair (TEVAR) have been applied successfully for reinterventions after coarctation repair [9,10] and are currently recommended as the first-line therapeutic approach in the European Society of Cardiology (ESC) guidelines [11], a significant number of patients may not be applicable for endovascular approaches due to anatomical factors or graft infections. Thus, open surgical repair remains an important cornerstone in the therapeutic management of long-term complications after coarctation repair [6]. Consequently, open descending aorta replacement as a redo operation after initial surgical coarctation repair must be part of the repertoire of aortic surgery centers of excellence even today. However, surgical experience and long-term follow-up data concerning this overall rare entity in the spectrum of aortic reoperations is still highly limited. Consequently, the main objective of this study was to close the gap in the current evidence-based knowledge on the outcome of open surgical descending aorta replacement in patients with complications after coarctation repair. As this is an overall rare entity, the evidence base for the outcome of open surgical descending artery replacement after coarctation repair is still insufficient to date. Nonetheless, this information is of importance for the clinician in the decision-making process for an endovascular versus surgical approach for patients with marginal applicability of endovascular techniques. Here, we provide an analysis of surgical techniques applied in our center as well as the short- and long-term results of open surgical replacement of the descending aorta as reoperations after coarctation repair.

## 2. Methods

A total of 650 consecutive cases of open surgical replacement of the descending thoracic aorta performed in our center from 2003 to 2023 were screened for patients who had previously undergone aortic coarctation repair by either patch aortoplasty or interposition grafting. Thereby, 25 cases were identified and analyzed in a descriptive retrospective single-center study with prospective follow-up (Supplemental Figure S1).

### 2.1. Preoperative Diagnostics and Indication for Surgery

Patients were referred to our center either from peripheral institutions with acute or chronic symptoms of late repair failure or with asymptomatic complications diagnosed during routine follow-up in our center for adults with congenital heart diseases or an associated outpatient clinic. For confirmation of the diagnosis and surgical planning, contrast-enhanced computed tomography or magnetic resonance tomography were performed as applicable considering the patient age and renal function. All patients received a preoperative transthoracic echocardiogram and left heart catheterization with selective coronary angiography was performed in all patients over the age of 40 years. The indication for surgery was evaluated for each patient individually taking into consideration the specific anatomy as well as cardiovascular and non-cardiovascular comorbidities.

### 2.2. Surgical Technique

To establish extracorporeal circulation, the femoral artery and vein were accessed via a vertical incision in the groin and cannulated prior to performing the thoracotomy. For isolated descending aorta replacements as redo operations after prior coarctation repair, a left-sided oblique thoracotomy in the 5th to 7th intercostal space starting at the angulus inferior of the scapula was used. The incision was expanded caudally by 10–15 cm depending on the extent of the planned repair. Afterwards, the M. latissimus dorsi and M. serratus anterior were divided and access to the left pleural cavity was gained during apnea. If encountered, adhesions resulting from the preoperation were carefully dissected and resolved to prepare the required cross-clamp sites. The targeted aortic section and, if necessary, the subclavian artery and phrenic nerve bundle were secured by vessel loops. In cases requiring clamping proximal to the left subclavian artery, the N. laryngeus recurrens was identified and carefully dissected free. After completion of the preparation of the situs, full heparinization based on body weight with a dose of 400–500 IU/kgBWT (body weight) was applied and extracorporeal circulation was initiated when an activated clotting time of >450 s was reached. The aorta was cross-clamped proximal and distal to the target segment, and the aorta including the patch or interposition graft material was incised and resected. If back-bleeding from intercostal arteries was noted, intercostal arteries were ligated with the exception of one case (patient No. 16), in which a large longitudinal aortic resection was necessary and intercostal artery reinsertion was performed using the button technique as previously described [12]. The proximal prosthesio-aortic anastomosis was completed and the clamp was moved distally onto the graft. Afterwards, the distal anastomosis was completed and both clamps were opened after careful deairing. After achieving hemostasis, the patient was weaned from extracorporeal circulation and the thoracotomy was closed in standard fashion. In one case (patient No. 25), concomitant aortic arch replacement was necessary due to involvement of the distal aortic arch and arteria lusoria in the patch aneurysm. In this case, the frozen elephant trunk procedure was performed via median sternotomy as previously described [13,14].

### 2.3. Data Curation and Follow-Up

Patient characteristics, intraoperative parameters, and postoperative complications were collected continuously in our institutional database. For long-term follow-up, regular cross-sectional imaging and outpatient clinic consultations were made in intervals of 6 months to 2 years depending on the time since the operation and temporal dynamics of the findings. Data were accessed and screened retrospectively and processed for this descriptive study.

### 2.4. Study Definitions

The following standardized definitions were used to facilitate a consistent assessment of the postoperative outcome. 

The necessity of re-intubation or non-invasive ventilation after previous spontaneous breathing was defined as postoperative respiratory failure. Diagnostic laryngoscopy was performed in all patients showing clinical signs of postoperative dysphagia or dysphonia. If new hypomobility or immobility of the left vocal chord was found, the diagnosis of left vocal chord paraparesis was made. Postoperative sepsis was defined according to the third international consensus definitions for sepsis and septic shock (Sepsis-3) [15]. Perioperative stroke was defined as any new symptomatic cerebral ischemia or bleeding and was diagnosed by clinical assessment and cerebral computed tomography or magnet resonance tomography. Paraplegia and paraparesis were diagnosed clinically. If the routine physical examination revealed a suspicious finding for paraplegia or paraparesis, this diagnosis was verified by a neurologist’s consultation. If the distribution of deficits detected in the clinical examination remained inconclusive with regard to a potential ischemic etiology, further diagnostic imaging in the form of a cranial or spinal CT was performed to rule out potential differential diagnoses such as paraplegia or paraparesis of cerebral origin. Spinal cord-associated neurological deficits were considered transient if they had regressed by the time of discharge and were otherwise defined as permanent. Acute kidney failure was defined as a three-fold increase in serum creatinine or urine output of less than 0.5 mL per kg body weight per hour for 24 h [16]. 

### 2.5. Statistical Analysis

Statistical analysis was performed using IBM SPSS Statistics 28 (IBM Corp. Armonk, NY, USA, 1989, 2021). Continuous variables were tested for normal distribution using the Kolmogorov–Smirnov test. Normally distributed data are given as mean ± standard deviation (SD). For non-normally distributed data, the median and interquartile range (Q1–Q3) are given. The Kaplan–Meier survival estimates were used to analyze overall survival and freedom from reintervention.

## 3. Results

Of the 650 consecutive descending aorta replacement procedures performed in the study period, 25 patients (3.8%) had undergone previous surgical aortic coarctation repair and were included in the descriptive study. In all of these cases, late complications of the initial coarctation repair were the main indication for surgical aortic reintervention. At 68% (n = 17), the majority of the patients were male, and with a mean body mass index of 25.6, the patients were, on average, mildly obese (Table 1). 

Concerning the concomitant congenital cardiovascular anomalies, bicuspid aortic valve morphology (32%, n = 8), persisting ductus arteriosus (12%, n = 3), and arteria lusoria dextra (8%, n = 2) were the most frequent abnormalities (Table 2). Coarctation repair was the first cardiosurgical procedure in the surgical history of all the patients. In the vast majority of the patients, coarctation repair was performed as an isolated primary operation, while two cases of simultaneous concomitant ligation of a persisting ductus arteriosus were registered. In 24% (n = 8), at least one cardiosurgical procedure was performed between the initial coarctation repair and descending aorta replacement. Among those, the majority of the operations addressed the aortic valve and the ascending aorta (Table 2). In these cases, the indication for aortic valve or ascending aorta replacement were predominantly concomitant abnormalities in the form of bicuspid aortic valve morphology leading to aortic valve insufficiency or concomitant aneurysm of the ascending aorta. In two patients (8%), two intermittent procedures were performed, making the descending aorta replacement the fourth cardiosurgical operation for these patients. The mean time between the coarctation repair and redo descending aorta replacement was 26.3 ± 9.9 years.

**Figure 1:**
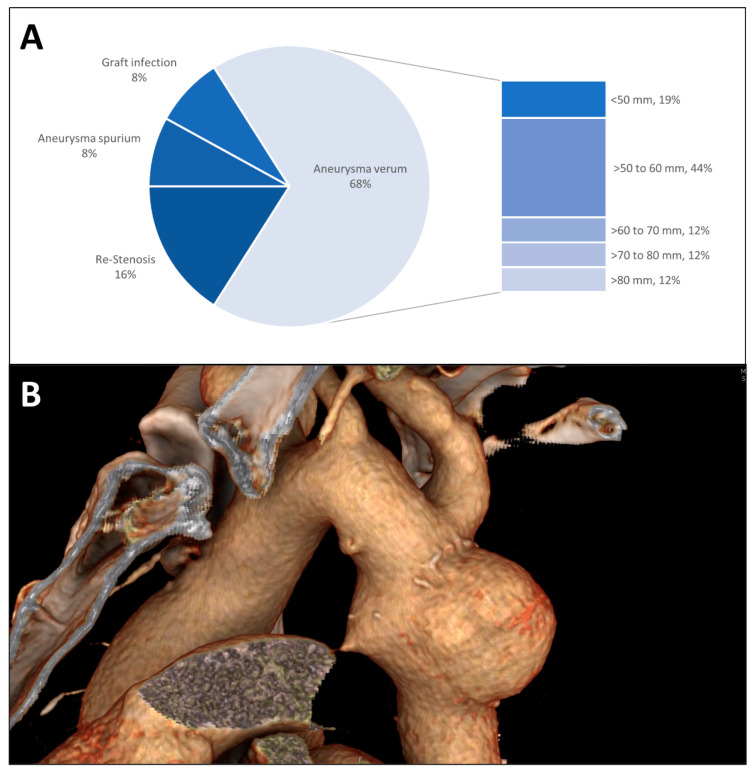
(**A**) Indication for surgery is shown in a pie chart; the group of true aneurysms is further divided by the maximum diameter at the time of the descending aorta replacement. (**B**) Three-dimensional CT radiographic reconstruction of a patch aneurysm after coarctation repair showing the typical spherical morphology and proximity to the left subclavian artery.

As an indication for descending aorta replacement, true aneurysm formation was the most common (Figure 1A). True aneurysms typically showed a spherical morphology spatially limited to the initial repair (Figure 1B). The median diameter of the true aneurysms was 60 mm (IQR 49 mm–70 mm) with 44% of the aneurysms measuring between 50 mm and 60 mm. Re-coarctation was the second most common indication for descending aorta replacement as an aortic redo procedure especially after initial interposition grafting followed by aneurysma spurium formation and graft infection. 

With the diagnosis and indication for surgery usually made during routine follow-up, the vast majority of the redo descending aorta replacements after initial coarctation repair were performed as elective operations (Table 3). However, one emergency operation was performed (patient No. 14) for the contained rupture of a spurious aneurysm after initial patch repair. 

**Figure 2:**
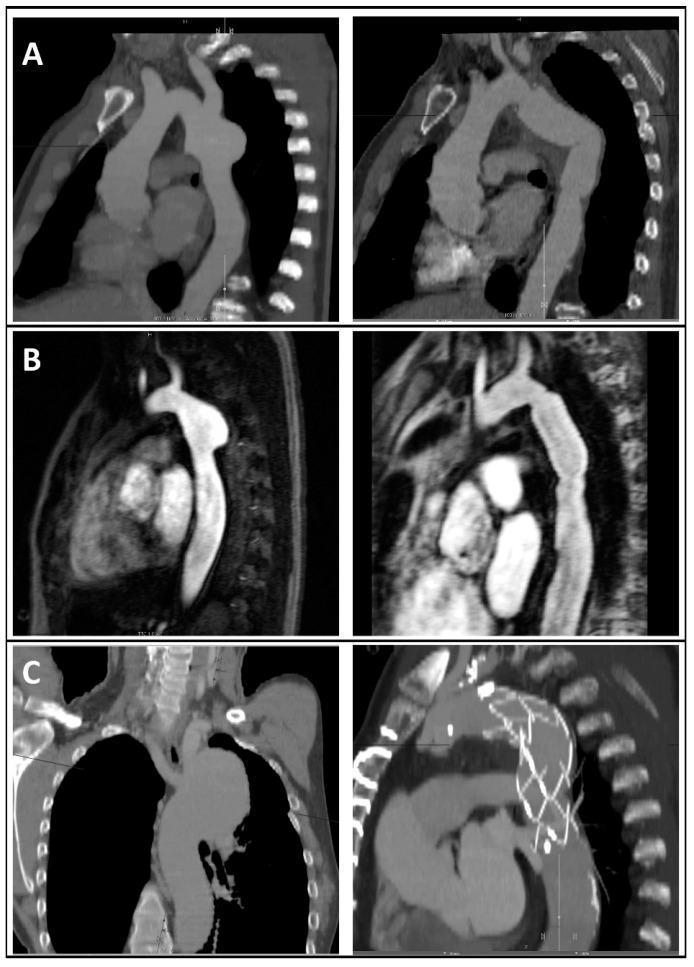
CT angiographic (**A**,**C**) or MR angiographic (**C**) reconstructions of different aneurysm morphologies after coarctation repair (**left**) and postoperative images after reoperation (**right**) showing descending aorta replacement and subclavian artery reinsertion in the prosthesis (**A**), isolated descending aorta replacement (**B**), or frozen elephant trunk implantation (**C**).

The procedure for all the redo cases except one was an isolated descending aorta replacement (Figure 2A,B). Polyethylene terephthalate-based Dacron^®^ double velour grafts were chosen for the isolated descending aorta replacements without graft infection (88%, n = 22). For the cases of graft infection, either a homograft or a silver-coated graft was used. For the one case of frozen elephant trunk implantation, the Jotec E-vita open neo^®^ hybrid prosthesis was used (Figure 2C). 

The operation times are shown in Table 4. In 64% of the cases (n = 16), clamping proximal to the left subclavian artery in aortic zone 2 was performed.

Regarding the short-term postoperative outcomes, left vocal chord paraparesis was identified as the most common complication followed by the need for rethoracotomy due to postoperative bleeding and respiratory failure requiring long-term ventilation and tracheostomy in two cases (8%, Table 5). 

In contrast, none of the patients had sepsis or wound infection and there were no cases of permanent neurological impairment. One patient showed transient paraparesis with full neurological recovery after two postoperative days.

**Figure 3:**
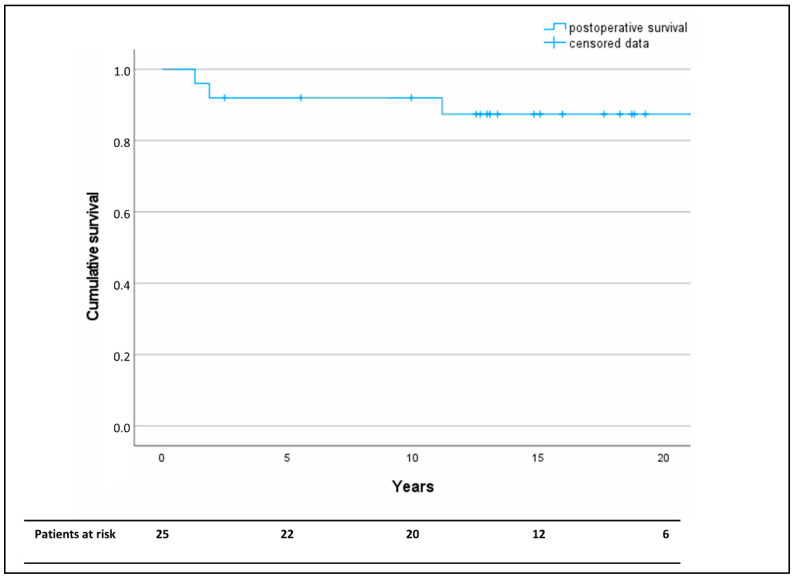
Kaplan–Meier analysis of long-term overall postoperative survival after descending aorta replacement as a redo operation following coarctation repair. Censored data are marked by horizontal lines.

In-hospital, 30-day, and one-year mortality was 0%. The Kaplan–Meier analysis revealed a survival of >80% at the median follow-up-time of 15 years (Figure 3).

## 4. Discussion

The results of this experience report can be summarized as follows: (1) Open descending aorta replacement after initial coarctation repair is an overall rare entity in the spectrum of adult aortic surgery. (2) Complications indicating the redo procedure occur late after initial repair and require long-term follow-up. (3) Redo operations after coarctation repair are often complex due to concomitant congenital cardiovascular comorbidities and/or cardiosurgical preoperations. (4) The early postoperative course is not infrequently complicated by bleeding or respiratory problems. (5) The short- and long-term survival after redo descending aorta replacement following coarctation repair were excellent.

Late complications after surgical repair of aortic coarctation are a well-known and not infrequent issue especially after patch aortoplasty or interposition grafting: von Kodolitsch et al. described an overall incidence of aneurysm formation following surgical coarctation repair in 9% of patients [17]. For patch aortoplasty, long-term complications requiring reoperation or -intervention are even more frequent and range up to 35% [18,19,20]. Thus, thorough long-term follow-up of patients after initial coarctation repair is essential. In our study population, the mean time between initial coarctation repair and redo descending aorta replacement was more than 26 years. This indicates that lifelong regular CT angiographic follow-up is of pivotal importance. Since complications can arise even several decades after initial repair, aortic surgeons may, even to this day, be confronted with complications of patch repair or interposition grafting, which were both widely discontinued in the early 2000s. In addition to the aneurysmal complications dominating in this study population, Zao et al. recently reported aortic re-coarctation after surgical aortic coarctation repair as another relevant complication especially in patients with associated aortic arch hypoplasia [21]. Further risk factors for aortic arch reoperations due to re-coarctation include a younger age at the initial repair operation and the need for prostaglandin E1 application [22].

Patients undergoing open descending aorta replacement not infrequently showed concomitant cardiovascular anomalies, of which aortic valve and ascending aorta pathologies constituted by far the largest part of the comorbidities. These findings are consistent with an earlier report by Bobylev et al., who showed a high association among coarctation of the aorta, aortic aneurysm formation, and bicuspid aortic valve morphology [8].

There is very little recent literature on the postoperative outcome after redo replacement of the descending aorta following coarctation repair. All case series available to date originate from the pre-endovascular period and showed highly unsatisfactory short- and long-term results. Knyshov et al. reviewed 48 patients with complications after coarctation repair. In this population, reoperation was performed in 30 patients. They reported a perioperative mortality after redo surgery following coarctation repair of 14% [7]. However, it should be noted that that study also included repeated patch aortoplasty (n = 6) and aortorrhaphy (n = 2), which have not been performed in our institution and thus are not included in this study.

Recurrent nerve paraparesis (24%, n = 6) and postoperative bleeding (20%, n =5) were among the most frequent short-term perioperative complications observed in our patient collective. These findings are consistent with previous reports by Ala-Kulju et al., who reported a series of 22 patients undergoing reoperation for aneurysm formation after coarctation repair. With a mean age of 25 years at reoperation, their patient population was distinctively younger compared to ours. They too describe recurrent nerve paraparesis and bleeding requiring rethoracotomy as the most frequent perioperative complications, at proportions of 36% and 32%, respectively [23]. In our study, the comparatively high rate of recurrent nerve paraparesis correlates with the high frequency of clamping proximal to the left subclavian artery. This phenomenon may be due to a shift in the surgical patient population over time with increasingly frequently used endovascular techniques, especially for uncomplicated and locally contained aneurysms. Thus, surgical reoperation became more and more the option chosen for patients not applicable for endovascular techniques, e.g., high proximity or involvement of the aortic arch and supra-aortic vessels, which may explain the comparatively high rates or clamping within the aortic arch zone 2.

Graft infections represent another complication after coarctation repair that cannot be addressed by endovascular techniques. In this study, two patients (8%) presented with graft infection after coarctation repair necessitating surgical reoperation. Both, a silver coated Dacron^®^ graft and a homograft replacement were used in this series with good short- and long-term survival outcome. In a comparative study for abdominal aortic infections, both prosthesis types have shown comparable effectiveness concerning containment of the surgical field and short- and mid-term survival [24].

## 5. Limitations

As this study is a retrospective single-center analysis, certain limitations apply including possible selection bias due to the analysis in a single center. Furthermore, although the patient population analyzed here is among the biggest samples with reports of this extremely rare entity, the number of patients was limited, leading to potential under- or overestimation of certain risks. The descriptive study design is additionally associated with possible confounding time- and practice-related effects. Data on the number of blood transfusions were not collected systematically, while the comparatively high rate of rethoracotomies due to bleeding may serve as an indirect parameter for increased perioperative blood loss.

## 6. Conclusions

While endovascular repair has become the gold standard for the management of isolated descending aortopathies including uncomplicated aneurysm formation after surgical coarctation repair, open descending aorta replacement still has to be part of the repertoire of aortic centers for patients unsuitable for the endovascular approach. Contrary to the unsatisfactory short- and long-term outcome of open surgical redo descending aorta replacement observed in early reports, the survival and morbidity has greatly improved over time. Thus, today, open surgical descending aorta replacement can be performed safely and with excellent survival outcomes even in the challenging subgroup of patients after previous coarctation repair, as shown in this more up-to-date analysis. Nonetheless, today, patients with complications after coarctation repair still represent highly challenging cases due to coexisting cardiovascular malformations or cardiovascular preoperations, which should be treated in specialized high-volume aortic centers.

## Figures and Tables

**Table 1:** Preoperative characteristics. BMI = body mass index.

Characteristics	N = 25
Sex (male)	17 (68%)
Age at operation (years)	45.4 ± 12.8
BMI (kg/m²)	25.6 ± 3.9
Marfan	1 (4%)
Arterial hypertension	13 (52%)
Hyperlipidemia	2 (8%)
Diabetes	1 (4%)
Coronary artery disease	0 (0%)
Chronic renal insufficiency	1 (4%)

**Table 2:** Surgical history. BAV = bicuspid aortic valve, CoA = coarctation of the aorta, VSD = ventricular septal defect, PDA = persistent ductus arteriosus.

Patient No.	Cardiovas. Comorbidities	Primary Preoperation [Age]	Secondary Preoperation [Age]	Tertiary Preoperation [Age]	Descending Replacement [Age]	Following Cardiovascular Operations
1	BAV, asc. aneurysm	CoA repair (interposition graft) [19 y]	Aortic valve replacement [30 y]		35 y	Composite replacement of the aortic valve an ascending aorta [37 y]
2	None	CoA repair (patch plasty) [22 y]			36 y	None
3	VSD, PDA	CoA repair (patch plasty), PDA ligation, pulmonary artery banding [0 y]	VSD closure (patch) + pulmonary debanding [2 y]	Futile balloon dilatation [18 y]	18 y	None
4	None	CoA repair (interposition graft) [23 y]			54 y	None
5	BAV	CoA repair (patch plasty [14 y]			40 y	None
6	BAV	CoA repair (interposition graft) [22 y]	Aortic valve replacement [31 y]		45 y	
7	BAV, anomalous pulmonary venous connection, PDA	CoA repair (patch plasty), PDA ligation [10 y]			38 y	None
8	None	CoA repair (patch plasty) [0 y]			20 y	None
9	None	CoA repair (patch plasty) [26 y]	Aortic valve replacement [61 y]	Axillo-femoral bypass [63 y]	63 y	None
10	BAV	CoA repair (patch plasty [19 y]			36 y	None
11	None	CoA repair (patch plasty) [35 y]			62 y	None
12	None	CoA repair (patch plasty) [14 y]	Aortic valve replacement [35 y]		40 y	None
13	None	CoA repair (patch plasty) [23 y]			50 y	None
14	None	CoA repair (patch plasty) [13 y]			42 y	None
15	Arteria lusoria dextra	CoA repair (patch plasty) [25 y]	Lusoria artery transposition [42 y]		42 y	None
16	None	CoA repair (patch plasty) [30 y]			47 y	None
17	None	CoA repair (patch plasty) [45 y]			71 y	None
18	BAV, aortic arch hypoplasia	CoA repair (patch plasty) [16 y]	David Procedure, ascending aorta replacement [40 y]		40 y	None
19	None	CoA repair (patch plasty) [20 y]			75 y	LICA replacement (vein graft) [69 y]
20	None	CoA repair (patch plasty) [16 y]			44 y	None
21	None	CoA repair (interposition graft) [23 y]			55 y	Carotido-subclavian bypass, TEVAR
22	BAV, PDA	CoA repair (patch plasty) [20 y]	Aortic valve replacement [29 y]		57 y	None
23	None	CoA repair (interposition graft) [12 y]			63 y	None
24	None	CoA repair (patch plasty) [21 y]			44 y	None
25	BAV, Arteria lusoria dextra	CoA repair (patch plasty) [34 y]			47 y	None

**Table 3:** Urgency.

Characteristics	N = 25
Elective	22 (88%)
Urgent	2 (8%)
Emergent	1 (4%)

**Table 4:** Intraoperative characteristics.

Characteristics	N = 25
Operation time (minutes)	249.2 ± 54.35
Bypass time (minutes)	88.2 ± 40.5
Cross-clamp time (minutes)	50.5 ± 20.5
Ventilation time (hours)	12.4 (10.2–19.5)
Clamping proximal to left subclavian artery	16 (64%)
Intercostal artery reinsertion	1 (4%)

**Table 5:** Short-term postoperative complications.

Characteristics	N = 25
Respiratory failure	3 (12%)
Tracheostomy	2 (5%)
Left vocal cord paraparesis	6 (24%)
Sepsis	0 (0%)
Wound infection	0 (0%)
Bleeding requiring rethoracotomy	5 (20%)
Stroke	0 (0%)
Temporary paraplegia/paraparesis	1 (4%)
Permanent paraplegia/paraparesis	0 (0%)
Acute kidney injury	1 (4%)
Dialysis	0 (0%)

## Data Availability

Data used in this study are available from the corresponding author upon request.

## Supplementary Materials

The following supporting information can be downloaded at https://www.mdpi.com/article/10.3390/jcm13185345/s1, Figure S1: study flow chart.
