# Supplementary figures and images for: The Fate of the Aorta after Coarctation Repair: Open Surgical Replacement of Descending Aorta in a High-Volume Unit
Source: J Clin Med. 2024 Sep 10;13(18):5345. doi: 10.3390/jcm13185345 (PMC11432251; doi:10.3390/jcm13185345)

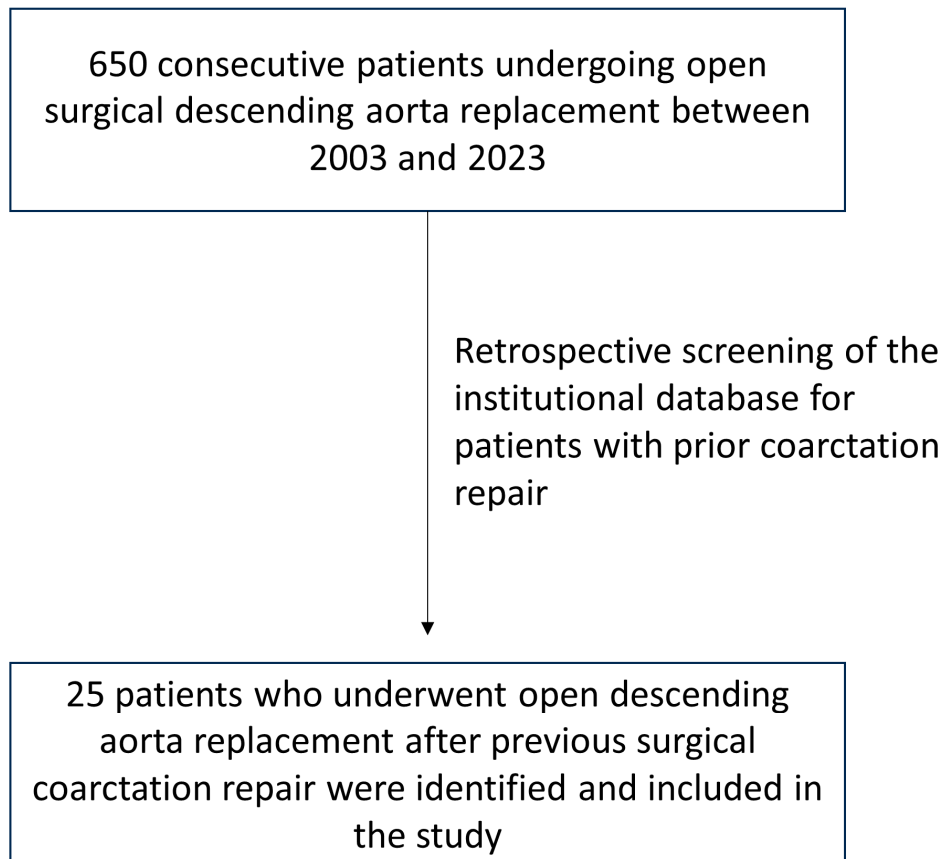

**Figure S1.** study flow chart.

Supplement: Supplementary file 1 [file jcm-13-05345-s001.zip › jcm-3176616-supplementary.pdf]
